# News and Public Opinion Multioutput IoT Intelligent Modeling and Popularity Big Data Analysis and Prediction

**DOI:** 10.1155/2022/3567697

**Published:** 2022-02-12

**Authors:** Hao Yan

**Affiliations:** School of Education and Modern Art, Shangqiu Institute of Technology, Shangqiu, Henan 476000, China

## Abstract

Based on the news and public opinion multioutput Internet of Things architecture, this article analyzes and predicts its popularity with big data. Firstly, the model adopts a three-tier architecture, in which the bottom layer is the data layer. It is mainly responsible for the collection of the terminal sensor data of the Internet of Things, and it uses intelligent big data as the data warehouse. Secondly, the computing layer on the data layer mainly provides the computing framework. Using the open-source SQL query engine, a cluster environment based on memory computing is constructed to realize the parallelization of data computing. It is used for interactive operations between the system and users. It receives and forwards the query requests submitted by the client browser, transmits them to the server cluster for execution, and displays the results in the browser. The end is displayed to the user. After that, combined with the needs of the development and application of news and public opinion big data, the data collection process was analyzed and designed, and the distributed data collection architecture was built. The intelligent Internet of Things was adopted for data storage, the data storage structure was analyzed and designed, and the data storage structure was designed to avoid catching. The repeat check algorithm is used to repeatedlystore the obtained page data. At the same time, according to the analysis of the business needs of the news and public opinion information platform, the overall functional structure of the platform was designed. The database and platform interface were designed in detail. The simulation results show that the model realizes the statistical query of the collected sensor alarm information and historical data on the user system, combines the historical operating data to analyze the relationship between the supply/return water temperature of the heat exchange station and the outdoor temperature, and realizes chart visualization of data analysis.

## 1. Introduction

With the development of modern information technology, emerging technologies, such as the Internet, cloud computing, and big data, have been widely used in various fields of social economy. The sensor transmission speed of the Internet of Things is very fast, and the application of a large number of sensor devices will definitely lead to a substantial increase in the output of news and public opinion data. The Internet of Things and big data are closely related, and the data generated by sensors can also be processed by the big data platform [1, 2]. News and public opinion Internet of Things big data is different from Internet big data [3]. In addition to the general characteristics of big data, they also have strong relevance and timing. Therefore, the traditional Internet big data processing methods are not fully applicable. New solutions need to be designed specifically to properly analyze the Internet of Things data and extract more important information from the Internet of Things monitoring equipment [3–5].

To improve the efficiency of the analysis of news and public opinion IoT data, it is necessary to implement a low-latency query system based on the Hadoop data warehouse, which can handle a large number of concurrent query requests. A single query can return the results faster, thereby improving the efficiency of data processing and analysis [6]. With the increasing maturity of the wireless sensor network technology,the combination of RFID, other sensors and wireless communication technology can realize the information exchange between objects,as well as long-distance monitoring and management if connected to the Internet. The internet realizes human-to-human interaction with people as the object, and the Internet of Things will further realize the interaction between things and realize the concept of ubiquitous networks. Using distributed query strategies and memory-based computing methods, the main purpose is to quickly query the information in massive amounts of data, provide timely feedback to users, and improve query efficiency. For massive data, traditional processing methods are difficult to meet real-time requirements. Distributed technology has become a research hotspot in the field of big data [7–9].

Based on this, the system takes the news and public opinion as the research object and uses computer network and communication technology under the framework of the Internet of Things to store the news and public opinion-related information and deliver it to the users in a timely and accurate manner, realizing news and public opinion detection, management, and marketing network. The traditional news and public opinion detection technology has been improved to improve the efficiency of news and public opinion detection, personnel management, and business decision-making. In the process of platform construction, a data storage architecture based on Mysql and intelligent Internet of Things was designed, SpringMVC development framework was adopted. Spring Framework was used as the core container, and Dubbo was used as the distributed architecture of the entire platform.The platform system is powerful, safe and scalable, which can handle high concurrency and massive data storage.Dubbo chooses intelligent big data as a data storage tool and creates news, public opinion, and IoT big data based on an open-source memory computing engine. Query the system and use data mining algorithms to analyze the historical data of the operation of the heat exchange station in the news and public opinion detection.

## 2. Related Work

With the continuous increase in the popularity of big data research, the application fields of big data are becoming more and more extensive. Domestic internet companies use big data technologies, such as Hadoop, to handle PB-level data problems in data storage, data mining, and high concurrency. Using Hadoop-based architecture for data collection, data analysis, etc., major universities are also conducting academic research and applications in the big data environment. Under the general situation, big data has been widely used in internet finance, medical health, transportation, and communication operations for other aspects [10–12].

Losavio et al. [13] proposed a news and public opinion big data acquisition and analysis platform (IBDP) that integrates HDFS, Spark, intelligent big data, HBase, Flum, Sqoop, OpenStack, etc., suitable for the acquisition and analysis of news and public opinion data. Yigitcanlar et al. [14] proposed and developed a smart city system based on the Internet of Things using the Hadoop ecosystem and big data analysis technology, combined with Spark over Hadoop to achieve the efficiency of big data processing. Hossein Motlagh et al. [15] proposed the use of the Hadoop software environment, including data collection, data storage, data normalization and analysis, and data visualization components to realize the parallel processing of large heterogeneous data for IoT network security monitoring. Scholars have developed a farmland observation data management system based on the integration of wireless sensor networks that realize the automatic acquisition of a large amount of news and public opinion data. The system has been applied in the city for the related processing and analysis functions. Chin et al. [16] studied news and public opinion big data from the connotation of news and public opinion big data, the acquisition of news and public opinion big data, and the status quo of news and public opinion big data and combined the current internet technology and big data technology to look forward to news and public opinion big data.

Van Deursen and Mossberger [17] elaborated on the four key technologies of big data research: data collection and preprocessing, data storage, data analysis and mining, and data presentation and application, and gave the architecture diagram of big data collection, data warehouse, parallel storage architecture.In addition, they also introduced the high-availability technology of mass storage system, parallel computing, real-time computing, streaming computing, deep learning, data privacy protection technology, and other related technologies, and provided reference learning cases. According to the characteristics of massive data, the researcher designed the system structure of the massive data management system based on Hadoop and introduced the distributed storage and distributed computing of the massive data in detail, which has a strong reference value [18]. At present, there are many types of enterprise information management systems, and each system is managed independently, resulting in the wastage of resources and poor scalability of the system. In this backdrop, Lu designed a hierarchical storage system using the Hadoop key technology to design data mining function, data migration module, etc., to provide data mining and data migration system based on Hadoop architecture. It shows that with the continuous update of internet technology, the complexity of the news and public opinion management information system is also increasing. However, few news and public opinion management information systems can use all the functions of the internet and the emerging accurate news and public opinions and face the news and public opinion management information. The system's requirements for accurate news and public opinion and traditional management information systems require that the implementation of these systems is more complex. Some studies are based on the need of identifying accurate news and public opinions and realizing news and public opinion management by evaluating modern networks [19–21].

## 3. Based on News and Public Opinion Multioutput IoT Intelligent Modeling and Popularity Big Data Analysis and Prediction Model Construction

### 3.1. Intelligent IoT Hierarchical Nesting

**Figure 1:**
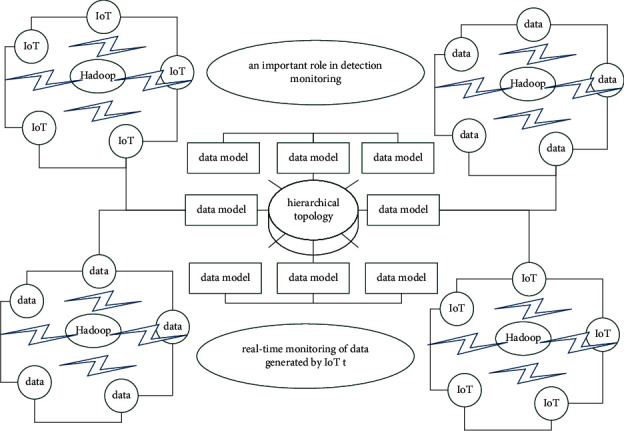
Smart IoT hierarchical topology.

At the intelligent IoT level, data analysis needs to move a subset of data to the data warehouse, and the speed of data analysis in Hadoop is very slow. However, with the development of SQL query engines, big data technology can already be used in business analysis scenarios. By building a data model in Hadoop or other databases, large-scale historical data accumulated and stored for a long time are used in the big data processing system for information mining [22].  Figure 1 is the hierarchical topology of the intelligent Internet of Things.

The data collected by the sensors play an important role in detection monitoring and data mining analysis. In addition to real-time monitoring of data generated by IoT terminal devices, it is also necessary to store historical data accumulated in the process of news and public opinion detection and provide real-time statistical query analysis and the function of generating data reports.

(1) $$\begin{array}{c} L\left(m,t\right)=\frac{l\left(m,t\right)}{N},\\ \psi \left(x\right)=\frac{f\left(x,d\right)-f\left(x,m\right)}{f\left(x,d\right)+f\left(m,d\right)}. \end{array}$$

In the data monitoring system of IoT devices, the sensor devices transmit the monitoring data to the data processing platform using various transmission methods, such as HTTP, TCP, and MQTT and store them in MySQL after parsing to provide real-time query support. The massive historical data adopts the intelligent big data storage warehouse to provide large-scale data support for the data mining and analysis of the system.

(2) $$\begin{array}{c} \chi \left(x,\lambda \right)=\left\{\begin{array}{cc} e^{-\lambda L\times \left(1+\lambda x^{2}\right)}, & \chi \geq \chi \left(x\right),\\ 0, & \chi <\chi \left(x\right), \end{array}\right.\\ L\left(y,g\right)=\sum_{i=1}^{n}y_{i}\left(g_{i}+\ln\;\times \sum_{j=1}^{n}\exp\left(g_{i}\right)\right). \end{array}$$

When performing statistical query and data mining analysis on historical data, a query engine based on memory calculation is used to improve the speed of the system query and analysis. At the application layer, the output of the big data platform layer is used for chart display and to build a web server platform to provide a visual interface for data display and analysis. The processing of data collected by the terminal equipment includes real-time monitoring, statistical query analysis, and data mining analysis.

(3) $$\begin{array}{c} \left\{\begin{array}{c} f\left(x\right)=\text{sign}\left\{\sum_{i=1}^{N}a_{i}y_{i}\times k\left(x_{i},x_{j}\right)-b\right\},\\ \text{sign}\left(x\right)=\left\{\begin{array}{c} -x>0,\\ x=0,\\ x\leq 0. \end{array}\right. \end{array}\right. \end{array}$$

The terminal collection system consists of wireless IoT sensing devices, gateways, and data storage servers. The terminal devices regularly transmit data to the gateway through a network protocol. The collected data is parsed by the gateway and stored in a unified format.

### 3.2. Multioutput Process of News and Public Opinion

**Figure 2:**
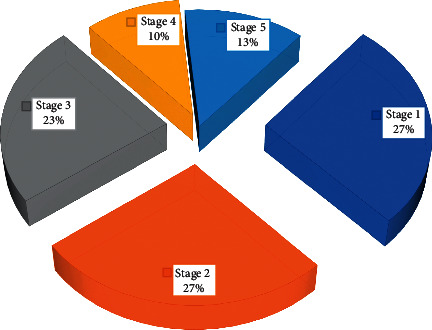
Multioutput fan chart of news and public opinion.

News and public opinion have different output data nodes, and the collected data information will also have different degrees of difference. It is necessary to unify the data access methods of all IoT application terminals and storage standards for different forms of data at different nodes and unify the storage and management of multisource data. At the same time, it is necessary to provide hybrid computing capabilities for multisource data to improve the efficiency of multisource data management and analysis. The web server receives the request submitted by the client browser and sends the query task to the Presto computing cluster, and finally, it returns the result of the query execution to the user and displays the result on the client browser in the form of a web page for the data analyst to provide a friendly visual interface. The front-end is mainly implemented with Ajax, JavaScript, jsp, CSS, html, and other technologies, and ECharts is used to realize chart visualization.  Figure 2 is a multioutput fan chart of news and public opinion.

When the coordinating node of the ZigBee network receives the single-point-sent data packet from itself or other devices, the network layer will continue to pass it on according to the program we set up in advance according to the requirements. If the target node is its neighbor, the data packet will be directly transmitted to the target node. If the target node is not its neighboring node, the coordinator node will retrieve the record that matches the destination address of the data to be transmitted and the routing table. If there is a match, the data will be transmitted to the next level network in the record; otherwise, the router will initiate a path search. When a node device receives an RREQ data packet, it will forward the data packet, in turn, and add the latest connection cost value. By analogy, the RREQ data packet follows all the connections it passes through and carries the sum of all connection costs until the RREQ data packet successfully reaches the destination device node. The routing node will select the one with the lowest connection cost among the received RREQ packets and send a route reply packet RREP to the source device. The RREP packet is a single-point sending packet that will return to the node device that sent the request along the path where the RREQ packet came from. After the path is established, the data packets can be sent and received along the established path. At this time, the node will send an RERR packet to all the node devices waiting to receive the data of this node to set the path as invalid. Each node will also update its routing table according to the received data packet.

### 3.3. Popularity Demand Analysis

In the computing structure of popularity, the data stream is composed of countless tuples. It is the smallest unit of data that contains many key-value pair data. The spout is the entrance to the data source. It provides many simple API interfaces, including sensor output API interfaces, website hits, messages from social networking sites, and logs of various applications. It converts the received data source into a tuple data stream and transmits the data to the next component specified. The main purpose of writing a spout program is to obtain data from the data source. Bolt is a function in the storm program. It is responsible for calculating and processing the input data stream. Its main functions include data filtering, data fusion, data calculation processing, and writing data to or reading data from the database. The terminal sensor device sends data to a unified data processing platform through the gateway so that each device can share and exchange data seamlessly. At the same time, it integrates the processing of real-time data and historical records and provides a unified operating environment for the application platform of the Internet of Things. Integrate demand analysis and solutions in a variety of application scenarios so that the system can meet the various needs of the users.

**Figure 3:**
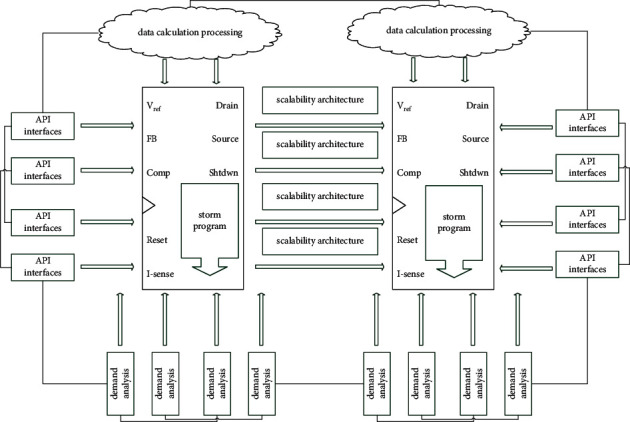
Popularity requires scalability architecture.

Figure 3 shows the popularity requirement scalability architecture. NewSQL mainly refers to the improved SQL database with scalability and superior performance. As it is an improvement and innovation based on the original SQL database, the NewSQL is compared to the original SQL database technology. Since it is an improvement and innovation of SQL technology, it still has the functions of traditional SQL, supports SQL queries, and meets the transactional and consistency requirements of the database queries. At the same time, the improved NewSQL database also has scalability and flexibility similar to NoSQL databases. HDFS supports the redundant backup storage of data blocks. As HDFS requires a combination of relatively low-cost small computers, these small computers are not highly reliable, and hence, HDFS is designed to be highly fault-tolerant. It is able to detect and respond to the failure of each machine node in time and ensure the stability of the system.

For this part of the data source, a variety of data acquisition interfaces need to be developed in the system, including the RS485 interface, Ethernet interface, AD conversion interface, and 24 V switch detection interface. By deploying data to HDFS, it can support large-capacity, high concurrency, and high-throughput big data computing tasks, while keeping file systems consistent across the nodes. An HDFS cluster must contain a name node NameNode (aka master node) and multiple DataNodes (aka slave nodes). Each slave node in the HDFS system is an ordinary or cheap computer. The name node can provide a naming service for each storage unit in the HDFS system, record and maintain the mapping information of the entire system data block, and receive requests to access HDFS from the corresponding client. Data nodes are mainly used to perform specific tasks, such as storing file blocks scheduled by the client and the NameNode. At the same time, HDFS has a client interface that interacts with the outside world. It mainly implements the external access requests to HDFS, including interacting with the NameNode to read file storage information, interacting with the DataNode to read the data in the HDFS system, and so on.

### 3.4. Design of Big Data Forecasting Function

**Figure 4:**
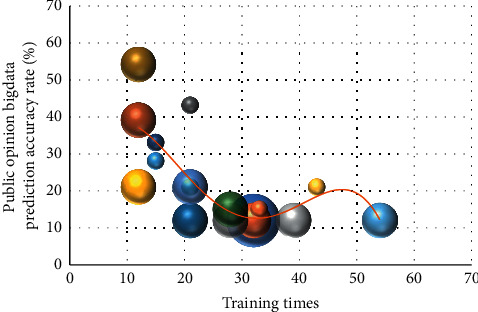
Distribution of prediction accuracy of public opinion big data.

The Hadoop system has many components, including HDFS, MapReduce, HBase, smart big data, Sqoop, Flume, Zookeeper, etc. It can run efficiently on the Linux platform. It supports multiple programming languages and has high reliability and fault tolerance. It can process the data reliably and efficiently and has relatively good scalability. It is used to store and process large amounts of data for analysis, and thus, Hadoop has become a very popular solution. When the Hadoop cluster becomes insufficient, it is usually improved by adding new computers or storage devices with common methods. The Hadoop ecosystem can perform distributed computing well, and the users can develop without knowing the details of the underlying storage. The data collection terminal mainly relies on wireless network sensor equipment for real-time data collection. The wireless node performs data collection after registering in the network. At the same time, the data is sent to the gateway through the MQTT protocol and Modbus bus at regular intervals. The data of multiple nodes are summarized. The collected data is preprocessed and sent to the relational database server in a unified format, and the application server side processes the data.  Figure 4 is the distribution of prediction accuracy of public opinion big data.

In streaming computing, the data continuously flows into the system. The streaming computing system analyzes and calculates the continuous data in real-time and quickly in the memory, and then, it feeds the results back to the user in real-time or stores them for subsequent queries. Traditional streaming computing systems are mostly designed based on event mechanisms, and the amount of data that can be processed is limited. However, new streaming computing technologies, such as S4, Storm, and Spark, are mainly oriented to the use of streaming processing. The NameNode enables the clients to quickly access the required blocks for regular operations, adopts block placement strategies and replication mechanisms to ensure data availability and durability, and allocates new block locations while maintaining the load balance of the cluster. The main task of the DataNode is to store data. When a new data request is stored in HDFS, it will be split into blocks with a fixed preconfigured size, stored in the DataNode, replicated a fixed number of preconfigured times, and stored in different nodes. The DataNodes and NameNode generally communicate by a heartbeat message mechanism every few seconds so that the NameNode can know which node is unavailable and other useful information about the node. The client directly communicates with the DataNode when accessing data. When the client forwards the request to HDFS, the NameNode, firstly, sends back the location of the block required by the client after verifying the relevant license, and then, the client directly commands the DataNode to execute the required block.

## 4. Based on News and Public Opinion Multioutput IoT Intelligent Modeling and Popular Degree Big Data Analysis and Prediction Model Application

### 4.1. Smart IoT Big Data Query

This paper uses ATMEGA32-16AU type single-chip microcomputer of ATLEL Company, which is a low-power and high-performance 8 bit AVR microprocessing chip that uses an RISC structure. The single-chip core contains a rich instruction set and up to 32 general-purpose registers with 32K bytes of in-system programmable flash, 2K bytes of SRAM, 1K bytes of EEPROM, and 32 general-purpose I/O interfaces to meet the needs of the system. The I/O interface of ATMEGA32 one-chip computer can visit through the IN and OUT order and carry on data transmission between 32 general registers and I/O interfaces. The address of the register from 0x00 to 0xlf can be directly addressed by CBI and SBI commands, and the value of a certain bit in the address can be detected by SBIS and SBIC.

**Figure 5:**
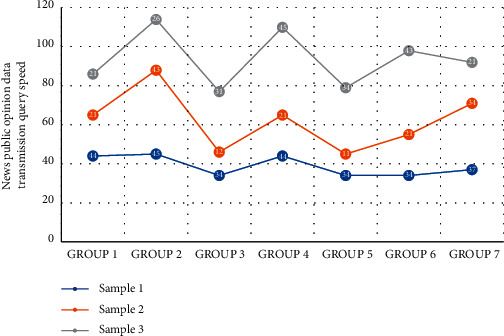
Distribution of news and public opinion data transmission query speed.

Figure 5 is the distribution of news and public opinion data transmission query speed. When querying, the embedded device sends a query command to the server. The server queries the database according to the command type and displays the data result on the embedded device via the coordinator, routing node, and terminal node. The entire architecture of the platform is mainly based on MySQL and MongoDB databases with Spring Framework as the core container combined with Zookeeper as the registration center and Apache Shiro as the authority authorization layer. My Batis is used as the persistence of the data access layer, and Redis is used as the cache database to improve the database access speed. In the data analysis layer, the Flume component can obtain a large amount of heterogeneous data in the HDFS storage system and supply it to the Hadoop offline batch processing system for analysis and processing. The processing results are written into the corresponding database. The Kafka component obtains the sensor network data stream and provides it to the Storm real-time data processing system for data analysis. The Storm system will process the analyzed data and write it into the corresponding database using the bolt component. Finally, there is the application layer. This layer queries and reads the data in the database according to the requirements of different applications or reads the processing and analysis results of the storm in real time.

### 4.2. Multioutput Platform Simulation of News and Public Opinion

**Figure 6:**
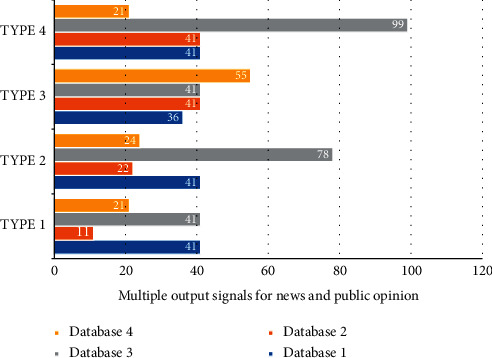
Multioutput signal distribution of news and public opinion.

When researching the news and public opinion information platform based on big data, the paper chooses the data storage mode combining Mysql and intelligent Internet of Things. Mysql is mainly used to store traditional business data, such as user information table, permission table, payment information table, agricultural material shop table, order information table, customer service information table, agricultural material evaluation information table, etc. However, the platform collects a large amount of user behavior data and a variety of types. Relational databases, such as Mysql can no longer meet the requirements. For alarm data and cloth length data with high real-time requirements, it is necessary to increase the frequency of data collection. The intelligent Internet of Things is stored in the BSON structure. Mass data storage has obvious advantages, and hence, the authors choose to use the intelligent Internet of Things to store the user behavior data. At the same time, for the business data on Mysql, a data backup is made in the intelligent Internet of Things. Among them, the data acquisition module uses various types of high-precision sensors and converts the collected analog signals into digital signals that can be identified and processed by the chip. In terms of communication designed in the thesis, the ZigBee communication technology is used for short-distance wireless transmission, and the GPRS and GSM communication networks are used for long-distance communication. The processing control module paper uses a single-chip microcomputer for processing information, a relay for controlling operations, a memory chip, etc. An integrated circuit board is made.  Figure 6 is the distribution of multiple output signals of news and public opinion.

**Figure 7:**
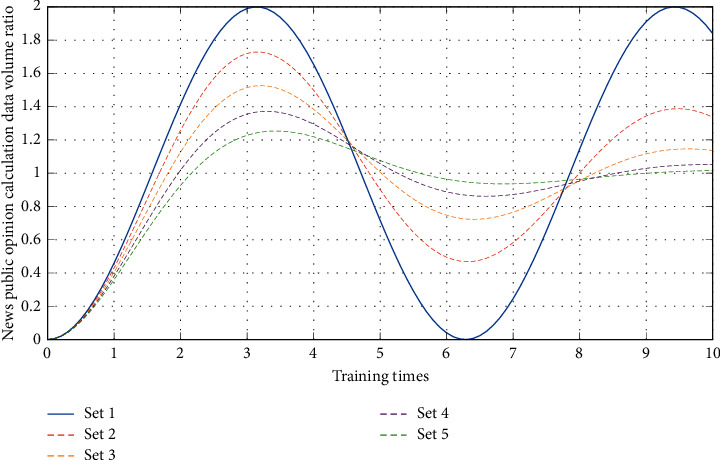
Distribution of data volume for news and public opinion calculation.

As the big data system mostly uses NoSQL database technology, a comparative study of this type of database technology is carried out so that a suitable database system can be selected according to the needs. There are many classification methods for NoSQL data inventory, and various classification methods may also overlap. If the database is compared according to the four types of data models, namely the key-value model, column model, document model, and graphical model, and the specific correlations of the four types of data models are shown in Figure 6. Therefore, it can be concluded that the increase in the amount of calculation data does not weaken Storm's computing power, which indicates that the data are effectively cached under the action of the Kafka component and ensures the smooth and efficient operation of the storm system.  Figure 7 is the distribution of news and public opinion calculation data.

When a device that has joined the network receives this sentence and the RejoinNetwork parameter is set to 0x00, NLME will send an NLMEJoIN. The confirm statement and the parameter value is INvALI. When a device that is not currently joining any network receives this sentence and the RejoinNetwork parameter is set to 0xol, the device will try to join the network specified by ExtendPANId, and then, NL dirty will issue MLME-AsSOCIATE. CoorAddress parameter setting is an address determined according to the situation of the router. This statement defines the initialization of the upper-layer device, allowing the upper-layer device to start a new ZigBee network and use itself as the coordinator. The Beaconorder parameter in the code represents the command for the network beacon formed by the upper layer. Super deorder means the command for the network superframe formed by the upper layer. BatteryLifeExtension means that if the value of this parameter is TRUE, NL will request coordination.Otherwise, NL will request that the coordinator does not support the battery life extension mode. The dynamic data sources of the warp knitting machine are diverse, including the warp let-off PLC of the warp knitting machine, electric meters, and various sensors. If a semifunctional node receives this sentence, NLME will send out an NLME-NETw0RK-FORMAT10 N whose parameter status is set to REQUEST confirm statement. The network cannot be established at this time. If a coordination node receives this sentence command, the device will be initialized as a coordination.

### 4.3. Example Application and Analysis

**Figure 8:**
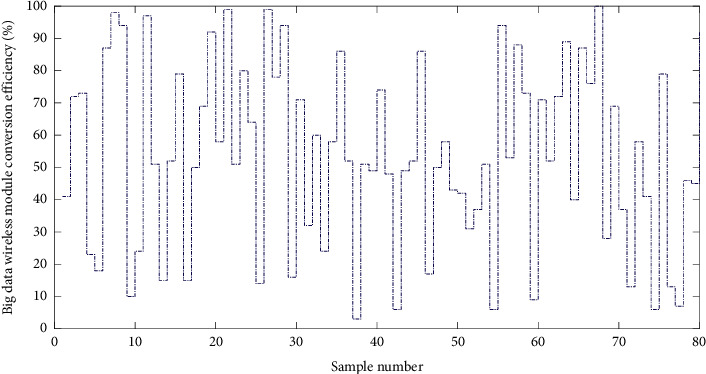
Conversion efficiency distribution of big data wireless modules.

Considering the above factors, this paper chooses the 10T-NODE2530 module. The sensor network node provided by this module is very complete. It supports the Zidie 2007 Pro wireless communication protocol very comprehensively. The node uses the CC2530 chip based on TI SoC. The chip's FLASH capacity is 256I (B, the module uses a standard interface for expansion, which can be expanded according to different application requirements because HBase runs on Hadoop, and hence, one needs to build a Hadoop cluster first. The cluster consists of four machines. It consists of four nodes. Among them, Node1 is the NameNode node, and the other nodes are the DataNode nodes. The wireless module is connected with the server using the RS232 level interface, and the serial port converter is connected to the upper computer. The serial port converter adopts the six-in-one multifunction of Technology Co., Ltd. The serial port module CP2102, USB, TTL, RS232, and RS485 four levels can be switched using the switch to realize the six serial conversion functions of USB to TTL, USB to 232, USB to 485, TTL to 232, TTL to 485, and 232 to 485.  Figure 8 shows the conversion efficiency distribution of big data wireless modules.

**Figure 9:**
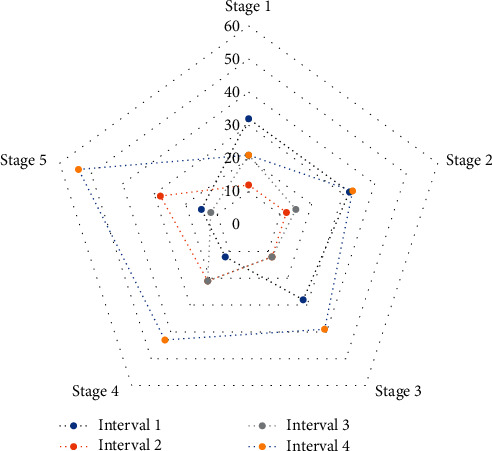
Distribution of news and public opinion serial port data acceptance rate.

GPRS specifies four forms of channel coding, namely CS-1, CS-2, CS-3, and CS-4, and their corresponding data rates are 9.05 kbps, 13.4 kbps, 15.6 kbps, and 21.4 kbps. The transmission rate is proportional to the wireless environment. It can be seen that the Cs-4 channel coding method has the highest requirements for C/I. At present, GPRS has been developed to support the multicoding mode and multislot technology fusion transmission, and its maximum speed can reach 171 kbps. The system software structure consists of a data source, query engine, and application server. The specific structure of the data query framework is shown in the text, where each component can be multiple in the system. Using MySQL and smart big data as the connection data source, MySQL is mainly used as a real-time query database. Smart big data can be used as a data warehouse for storing historical data. In the cluster, the query plan is executed by Presto. Firstly, the data is converted from the serial port data into IP data, and then, it is sent out through the GPRS transmission system. The processing control module sends data to the GPRS module through the Rs232 serial port. The packet data will be encapsulated by an SGSN, and the data will communicate with the gateway support node GGSN via the GPRS backbone network after being encapsulated.  Figure 9 is the distribution of news and public opinion serial port data acceptance rate.

In this experiment, the GPRS long-distance wireless transmission module is selected. The module uses the design scheme of the built-in protocol stack of the module. There is no chip outside the module, and hence, the module has higher stability. It supports up to 4 network connections and can connect to the serial port. The received data is sent to 4 servers at the same time. The module is set with a keep-alive mechanism to ensure that the network connection will not be disconnected when there is no heartbeat packet. It supports remote configuration parameters that can be configured by sending AT commands via SMS. The commonly used method of measuring the distance between the two nodes is based on the difference in the arrival time. This method uses the sending node to send two signals with different propagation speeds at the same time. Firstly, the receiving node calculates the difference between the arrival times of the two signals. Secondly, it combines their propagation speeds to calculate the distance between the two nodes. When querying in the same data table that stores the data records, the system time-consuming will increase with the increase of the query data. When the number of records in the data storage is different but the number of records in the query is the same, the system response time will increase as the total number of storage tables increases.

## 5. Conclusion

The article analyzes the specific sources of news and public opinion big data, the specific collection methods of data sources, and the methods of various database storage technologies, combined with wireless sensor network technology, open-source big data processing technology, and distributed data storage technology, and it proposes to solve big data. The article mainly works from two aspects of acquisition and storage. In terms of data acquisition, firstly, the application research on the sensor data acquisition network is carried out. Then, the sensor data acquisition system is designed and implemented. Finally, the system is used in news data acquisition and processing. The application was experimented with. In terms of data storage, the article mainly compares the database technologies under big data applications, designs the structure of the HBase data storage system when it is applied to news and public opinion sensor data storage, and conducts an experimental test on its storage performance. The system analyzed the actual needs of the project and the characteristics of big data of news and public opinion Internet of things, combined with the existing big data processing technology, and designed a system that can be used for quick query and analysis of big data of news and public opinion Internet of things. The system is implemented and tested on the Presto framework to achieve the effect of supporting the centralized storage and rapid query of massive news and public opinion detection data, as well as fit analysis and visual display based on historical data.

## Data Availability

The data used to support the findings of this study are available from the corresponding author upon request.
